# Cardiopulmonary resuscitation at operating room entry in acute aortic dissection type A patients: is surgery contraindicated?

**DOI:** 10.3389/fsurg.2024.1404825

**Published:** 2024-06-14

**Authors:** Hideki Isa, Taro Kanamori, Kazuki Miyatani, Masahiro Tsutsui, Ryohei Ushioda, Shota Yamanaka, Hiroyuki Kamiya

**Affiliations:** ^1^ Department of Cardiovascular Surgery, Kawaguchi Cardiovascular and Respiratory Hospital, Kawaguchi, Japan; ^2^ Department of Cardiac Surgery, Asahikawa Medical University, Asahikawa, Japan

**Keywords:** aortic dissection, preoperative cardiopulmonary arrest, cardiopulmonary resuscitation, out-of-hospital cardiac arrest, in-hospital cardiac arrest, return of spontaneous circulation

## Abstract

**Background:**

This study aimed to compare the short-term outcomes of surgical treatment for acute type A aortic dissection between patients undergoing cardiopulmonary arrest at the time of entry into the operating room and patients who received successful preoperative cardiopulmonary resuscitation before entering the operating room or patients who had cardiopulmonary arrest on the operating room table after entering the operating room without cardiopulmonary arrest. In the present study, we focused on the circulatory status at the time of entering the operating room because it is economically and emotionally difficult to cease intervention once the patient has entered the operating room, where surgeons, anesthesiologists, nurses, and perfusionists are already present, all necessary materials are packed off and cardiopulmonary bypass have already been primed.

**Methods:**

Twenty (5.5%) of 362 patients who underwent surgical treatment for acute type A aortic dissection between January 2016 and March 2022 had preoperative cardiopulmonary arrest. To compare the early operative outcomes, the patients were divided into the spontaneous circulation group (*n* = 14, 70.0%) and the non-spontaneous circulation group (*n* = 6, 30.0%) based on the presence or absence of spontaneous circulation upon entering the operating room. The primary endpoint was postoperative 30-day mortality. The secondary endpoints included in-hospital complications and persistent neurological disorders.

**Results:**

Thirty-day mortality was 65% (*n* = 13/20) in the entire cohort; 50% (*n* = 7/14) in the spontaneous circulation group and 100% (*n* = 6/6) in the non-spontaneous circulation group. The major cardiopulmonary arrest causes were aortic rupture and cardiac tamponade (*n* = 16; 80.0%), followed by coronary malperfusion (*n* = 4; 20.0%). Seven patients (50.0%) survived in the spontaneous circulation group, and none survived in the non-spontaneous circulation group (*P *= .044). Five survivors walked unaided and were discharged home; the remaining two were comatose and paraplegic.

**Conclusions:**

The outcomes were extremely poor in patients with acute type A aortic dissection who had preoperative cardiopulmonary arrest and received ongoing cardiopulmonary resuscitation at entry into the operating room. Therefore, surgical treatment might be contraindicated in such patients.

## 1 Introduction

Acute type A aortic dissection (AAAD) is a life-threatening disease that requires emergency surgical intervention to prevent fatal complications (1–3). The outcomes of surgical treatment for AAAD have improved significantly in recent decades but remain poor in patients with preoperative cardiopulmonary arrest (CPA) (3). However, if such patients survive in the initial period after surgery, the mid-term outcomes are reported to be acceptable (4), making the discussion on surgical indications in these patients challenging.

The main problem in discussing the surgical indications for patients with preoperative CPA due to AAAD is that the patient cohort is highly heterogeneous. Surgical results are acceptable when the patients require only temporary and short-term cardiopulmonary resuscitation (CPR). However, the results may be extremely poor when preoperative CPR is unsuccessful and the operation is initiated during ongoing CPR. Although scoring tools to predict mortality such as the Penn classification (5) and the German registry of acute aortic dissection type A (GERAADA) score (6) are available, to our knowledge, this issue has not been thoroughly investigated.

This study aimed to compare the short-term outcomes of surgical treatment for AAAD between patients undergoing CPA at the time of entry into the operating room and patients who received successful preoperative cardiopulmonary resuscitation before entering the operating room (OR) or patients who had CPA on the OR table after entering the OR without CPA. In the present study, we focused on the circulatory status at the time of entering the operating room because it is economically and emotionally difficult to cease intervention once the patient has entered the operating room, where surgeons, anesthesiologists, nurses, and perfusionists are already present, all necessary materials are packed off and cardiopulmonary bypass have already been primed.

## 2 Materials and methods

### 2.1 Patients

This study was approved by the Kawaguchi Cardiovascular and Respiratory Hospital institutional review board (2023-002). The requirement for obtaining oral and written informed consent was waived owing to the use of retrospective patient data.

Between April 2016 and March 2022, 362 patients underwent emergency surgical treatment for AAAD at our institution. Among them, 20 patients who required CPR before surgery were included in this study. The patients were divided into two groups, the spontaneous circulation group (SCG) and the non-spontaneous circulation group (NSCG), based on whether they had spontaneous circulation upon entering the OR. Return of spontaneous circulation (ROSC) was defined as the presence of a palpable pulse status, which persists after the initiation of extracorporeal membrane oxygenation (ECMO); however, no patient achieved ROSC after ECMO initiation in the present study.

### 2.2 Preoperative surgical risk evaluation

We calculated the Penn classification (5) and the GERAADA score (6) for all patients and compared the actual mortality rate to the calculated GERAADA score and the mortality rate estimated from their classification based on previous studies. All patients were classified according to the Penn classification, based on their condition at presentation, as follows: Penn class A indicated the absence of malperfusion or circulatory collapse, Penn class B indicated the presence of branch-vessel malperfusion, Penn class C indicated the presence of circulatory collapse, including coronary malperfusion, and Penn class B + C indicated the presence of both branch-vessel malperfusion and circulatory collapse.

### 2.3 Preoperative management

When AAAD was diagnosed, the patients were transferred to the OR immediately. However, the patients were transferred to the intensive care unit (ICU) when the OR was not prepared on time. If CPA occurred while waiting to enter the OR in the ICU or emergency room (ER), emergency thoracotomy or pericardial drainage through the subxiphoid incision was performed immediately.

### 2.4 Surgical technique

The patients underwent median sternotomy, and cardiopulmonary bypass (CPB) was promptly established via the femoral artery and right atrium. In a few patients undergoing CPR, the CPB was established directly from the ascending aorta. The left subclavian artery was used in one case. A left ventricular venting tube was inserted from the right upper pulmonary vein. When CPB was established via the femoral artery, the ascending aorta was clamped and incised, and selective antegrade cold blood cardioplegia was intermittently administered. Retrograde cold-blood cardioplegia was also administered in some patients.

Furthermore, at moderate hypothermia with a rectal temperature of 25°C, open distal anastomosis under circulatory arrest was performed. For brain protection, retrograde cerebral perfusion was performed in hemi-arch replacement only, and antegrade selective cerebral perfusion was added in cases of total or partial arch replacement. In principle, our strategy was entry site resection, including total arch replacement in patients with arch tears; however, hemi-arch or partial arch replacement was sometimes performed for life-saving reasons for patients with critical conditions. Aortic root replacement was performed if the intimal tear extending to the aortic root was difficult to repair.

### 2.5 Data collection and statistical analysis

All data were collected from electronic medical records. All statistical analyses were performed using EZR (7) (Saitama Medical Center, Jichi Medical University, Saitama, Japan), a graphical user interface for R (The R Foundation for Statistical Computing, Vienna, Austria); it is a modified version of the R commander designed to add statistical functions frequently used in biostatistics. Values are expressed as means ± standard deviations for continuous variables and *n* (%) for categorical variables. Continuous variables were analyzed using the *t*-test, and categorical variables were evaluated using the chi-square test (Fisher's exact test if *n* < 5). The level of significance was set at *P *< .05.

### 2.6 Outcomes

The primary endpoint was postoperative 30-day mortality. The secondary endpoints included in-hospital complications and persistent neurological disorders.

## 3 Results

There were 14 (70.0%) and 6 (30.0%) patients in the SCG and NSCG, respectively. The preoperative conditions of all patients are shown in Table 1. More than half of the patients in both groups were female, and the mean age in the SCG and NSCG was 72.5 ± 8.7 and 63.3 ± 15.6 years, respectively. In the SCG, the major cause of CPA was aortic rupture/cardiac tamponade (*n* = 12, 85.7%), followed by myocardial infarction (*n* = 2, 14.3%). In the NSCG, these affected four (66.7%) and two (33.3%) patients, respectively. In the SCG, all patients were intubated during CPR and underwent surgical treatment while intubated except for one patient who developed CPA after induction of anesthesia. In the NSCG, one patient developed CPA in the ICU and was immediately carried to the OR without ROSC or intubation. Two patients required preoperative ECMO without a palpable pulse, and both patients also required ECMO postoperatively. The remaining three patients presented with out-of-hospital cardiac arrest (OHCA) and entered the OR without ROSC. One of them was not intubated.

**Table 1:** Preoperative characteristics.

Characteristic	Overall (*n* = 20)	SCG (*n* = 14)	NSCG (*n* = 6)	*P* value
Age (year)	69.8 (±11.6)	72.5 (±8.7)	63.3 (±15.6)	.11
Male sex	6 (30.0)	3 (21.4)	3 (50.0)	.30
OHCA	13 (65.0)	9 (64.3)	4 (66.7)	1
IHCA	9 (45.0)	7 (50.0)	2 (33.3)	.64
Causes of CPA				
Aortic rupture/cardiac tamponade	16 (80.0)	12 (85.7)	4 (66.7)	.60
Myocardial infarction	4 (20.0)	2 (14.3)	2 (33.3)	1
ECMO support	2 (10.0)	0	2 (33.3)	.071
Emergent thoracotomy	2 (10.0)	2 (14.3)	0	1
Subxiphoid incision	1 (5.0)	1 (7.1)	0	1
GERAADA predicted mortality (%)	49.1 (±15.1)	48.6 (±15.0)	50.5 (±16.6)	.81
Penn classification				
Penn Class C	18 (90.0)	13 (92.9)	5 (83.3)	.52
Penn Clacc B-C	2 (10.0)	1 (7.1)	1 (16.7)	.52

Patients who achieved ROSC after presenting with OHCA and subsequently presented with IHCA were counted in both groups.

CPA, cardiopulmonary arrest; ECMO, extracorporeal membrane oxygenation; GERAADA, German registry of acute aortic dissection type A; IHCA, in-hospital cardiac arrest; OHCA, out-of-hospital cardiac arrest.

No significant differences were observed in operative time, CPB time, aortic cross-clamp (ACC) time, hypothermic circulatory arrest (HCA) time, extent of distal procedures, concomitant surgeries, including root replacement, or bleeding amount between the two groups (Table 2).

**Table 2:** Perioperative details.

Characteristic	Overall (*n* = 20)	SCG (*n* = 14)	NSCG (*n* = 6)	*P* value
Hemi or partial arch replacement	17 (85.0)	12 (85.7)	5 (83.3)	1
Concomitant procedure				
Aortic root replacement	7 (35.0)	5 (35.7)	2 (33.3)	1
CABG	3 (15.0)	2 (14.3)	1 (16.7)	1
Arterial cannulation site				
Femoral artery	14 (70.0)	11 (78.6)	3 (50.0)	.30
Ascending aorta	5 (25.0)	2 (14.3)	3 (50.0)	.13
Left subclavian artery	1 (5.0)	1 (7.1)	0	1
Operating time (min)	344 (±83.9)	370.2 (±71.4)	293.3 (±99.9)	.065
CPB time (min)	196.5 (±60.2)	202.5 (±60.3)	182.5 (±63.1)	.51
ACC time (min)	148.8 (65.7)	155.0 (±64.6)	134.2 (±72.0)	.53
HCA time (min)	38.5 (±17.6)	42.3 (±19.3)	29.7 (±8.3)	.15

ACC, aortic cross-clamp; CABG, coronary artery bypass grafting; CPB, cardiopulmonary bypass; HCA, hypothermic circulatory arrest; ROSC, return of spontaneous circulation.

Postoperative mortality and morbidity rates are presented in Table 3. “Stroke” in the complication includes fatal and non-fatal strokes. In the SCG, seven patients (50.0%) survived 30 days postoperatively; however, none survived in the NSCG (*P *= .044). In addition, no significant differences were observed in the causes of mortality or postoperative complications. Five survivors in the SCG could walk unaided and were discharged home (Table 4); two other patients were comatose and paraplegic, respectively, and were transferred to a rehabilitation hospital. In the NSCG, 4 patients (66.7%) had persistent neurological disorders. One patient in each group had involvement of the cerebral aortic arch branches by dissection, and both died of a fatal stroke. Univariate analysis showed that the significant risk factor for 30-day mortality in patients with AAAD having preoperative CPA was the CPA status at the time of entry into the OR (*P *= .044).

**Table 3:** Postoperative details.

Characteristic	Overall (*n* = 20)	SCG (*n* = 14)	NSCG (*n* = 6)	*P* value
30-day mortality	13 (65.0)	7 (50.0)	6 (100)	.044*
ICU stay (days)	4.6 (±2.9)	5.4 (±2.9)	2.8 (±2.6)	.084
Cause of mortality				
Bleeding	3 (15.0)	1 (7.1)	2 (33.3)	.20
Myocardial failure	2 (10.0)	1 (7.1)	1 (16.7)	.52
Massive stroke/hypoxic encephalopathy	6 (30.0)	3 (21.4)	3 (50.0)	.30
Sepsis	1 (5.0)	1 (7.1)	0	>.99
Visceral ischemia	1 (5.0)	1 (7.1)	0	>.99
Complications				
Dialysis	1 (5.0)	1 (7.1)	0	>.99
Stroke	9 (45.0)	5 (35.7)	4 (66.7)	.34
Pneumonia	1 (5.0)	1 (7.1)	0	>.99

ICU, intensive care unit.

* *P* value < 0.05 indicates statistical significance.

**Table 4:** Details of the survivors.

Patient	Sex	Age (year)	At the start of operation	After pericardiotomy	Reason for CPA	Neurological deficit	Discharge to	Modified ranking scale
1	Female	61	ROSC	−	Tamponade	Coma	Rehabilitation hospital	5
2	Female	74	ROSC	−	Tamponade	−	Home	0
3	Female	84	CPA	ROSC	Tamponade	−	Home	0
4	Female	77	CPA	ROSC	Tamponade	−	Home	0
5	Male	49	ROSC	−	Tamponade	paraplegia	Rehabilitation hospital	5
6	Female	80	ROSC	−	Coronary	−	Home	0
7	Female	79	ROSC	−	Coronary	−	Home	0

CPA, cardiopulmonary arrest; ROSC, return of spontaneous circulation.

### 3.1 Preoperative risk calculator/classification specialized to AAAD

All patients were classified to Penn Class C (*n* = 13 in the SCG, *n* = 5 in the NSCG) or B-C (*n* = 1 in SCG, *n* = 1 in NSCG) as a matter of course, and the GERAADA predicted mortality was 49.1% ± 15.1%, in all patients, 48.6% ± 15.0% in the SCG, and 50.5% ± 16.6% and the NSCG.

### 3.2 Primary and secondary outcomes

Thirty-day mortality was 50% (*n* = 7/14) in the SCG and 0% (*n* = 0/6) in the NSCG, as described above. Among surviving patients only in the SCG, 28.5% of patients (*n* = 2/7) suffered from a permanent neurological disorder; one with a coma and the other with paraplegia, but the remaining 5 patients were able to return to normal daily life.

## 4 Discussion

AAAD is a life-threatening disease reported to be responsible for death in 2.3% of patients with sudden cardiac arrest (8). Approximately 20% of patients with AAAD reportedly die before arriving at the hospital (9), and the in-hospital mortality rate remains high (22%) for patients who arrive at the hospital, as stated in a report from the International Registry of Acute Aortic Dissection (10). Several reports have stated that the postoperative in-hospital mortality rate of patients with AAAD with preoperative CPA is approximately 50% (11–13). Therefore, to make an appropriate surgical decision, objective assessments of the possibility of saving a life should be investigated, considering the patient's age, status of activities of daily living, and family wishes, which can be challenging.

Our institution has attempted to save lives by aggressively performing surgeries if the family wishes to do so, even if the preoperative condition of the patient is poor. In this report, we examined the factors that may decide treatment discontinuation in patients with AAAD and preoperative CPA.

Preoperative CPA has been frequently reported as a significant risk factor for operative mortality in AAAD (11–15). In this context, what are the characteristics of patients who survive a successful surgery? This study focused on whether patients exhibited spontaneous circulation upon entering the OR. Notably, patients with spontaneous circulation were more likely to survive; these patients achieved ROSC before entering the OR or had CPA after entering the OR.

We considered this result to be related to CPR duration. Several reports suggest that CPR duration is a prognostic factor for CPA; however, we could not examine the CPR duration in this study due to incomplete data, especially for patients exhibiting OHCA. Various cutoffs for CPR duration have been suggested in previous studies, such as 10 (16), 15 (11), and 20 (17) min; In reality, it would be extremely difficult to establish CPB within 20 min if the patient is in a state of ongoing CPR at the time of entering the OR. Therefore, the CPA status at OR entry should be considered when taking the decision to perform further surgery on the patient. Even if the patient's family expresses a strong desire for surgical treatment, careful consideration is warranted.

As shown in Table 1, the calculated GERAADA score of both groups was approximately 50%. This figure was equivalent to the actual mortality rate of the SCG, but significantly lower than that of the NSCG. Although several studies have reported the reliability of the GERAADA score (18, 19), this discrepancy may have occurred because the predictive score takes into account the presence or absence of preoperative CPR, but not its success. The Penn classification is another predictive tool that has already shown good efficacy in AADA (20, 21). Tien et al. reported the predicted mortality rates for AADA by Penn classification, and those of Penn class C and B-C were 15.8% and 40.0%, respectively (20), which differed from the results of this study. This classification is based on the presence or absence of branch-vessel malperfusion and circulatory collapse, and cannot reflect the most severe condition of preoperative CPA in its prediction. Even with the excellent tools already mentioned, it is difficult to predict mortality in the most critically ill patients, and the presence or absence of spontaneous circulation upon entering the OR, as reflected in this study, may be a potentially useful indicator.

In this study, the main cause for CPA in the SCG was cardiac tamponade, while in the NSCG one-third of the patients presented with myocardial infarction. Lin et al. reported that the survival rate of patients whose cause of CPA was myocardial infarction was lower than that of patients whose cause of CPA was cardiac tamponade. Their reasoning is as follows (12). First, cardiac tamponade during AAAD is usually induced by hemorrhagic leakage from a dissected ascending aorta, which accumulates in the pericardial space and compromises venous return, limiting cardiac output. Prompt management, including resuscitation and surgical rescue procedures, can efficiently reverse critical hemodynamics. On the other hand, myocardial infarction secondary to AAAD is caused by dissection or occlusion of a coronary artery induced by AAAD, and continuous cardiac massage or emergent cardiopulmonary bypass do not resolve coronary malperfusion. Therefore, myocardial ischemia persists. AAAD repair is a complex procedure. Even after successful surgical revascularization, irreversible myocardial damage may occur during prolonged ischemia. For these reasons, the mortality rate for AAAD complicated by myocardial infarction may be higher than that for AAAD complicated by cardiac tamponade. We did not find a significant difference in this study; however, we believe that AAAD complicated by cardiac tamponade and cardiopulmonary arrest should be treated aggressively to achieve better survival rates.

Two patients who underwent extracorporeal cardiopulmonary resuscitation (ECPR) using ECMO did not survive. Nakai et al. reported that seven patients with AAAD who underwent preoperative ECPR and achieved ROSC underwent immediate aortic repair; however, all patients died in the hospital, including the two patients with hypoxic encephalopathy who survived initially for more than 1 year (22). The authors stated that preoperative ECPR in patients with AAAD is not contraindicated; however, it is not recommended. In our experience, operative indications should be restricted in patients who do not achieve ROSC even after preoperative ECPR.

In this study, five of the seven survivors had no neurological deficits. However, Lee et al. reported that among 2,982 surgical cases of AAAD, 427 of 2,463 survivors had a permanent stroke or transient ischemic attack or paraplegia (14), indicating that 17.3% of all survivors had neurological deficits. In contrast, in our study, the neurological prognosis of survivors who exhibited preoperative CPA may not have been as poor. When it comes to surgical techniques, although there are some reports that subclavian cannulation is associated with fewer stroke complications than femoral cannulation (23, 24), we performed femoral artery cannulation except when a femoral artery was perfused by false lumen, calcification of a femoral artery was severe, or there was malperfusion of the lower extremities. Elbatarny et al. reported similar complication rates for both procedures (25), and, considering the simplicity of the procedure, femoral cannulation may be appropriate for the most severely ill patients after CPA, even if they are stable at the time of surgery. The ideal temperature in hypothermic circulatory arrest during AAAD repair is another controversial topic. Our surgical strategy—moderate hypothermic circulatory arrest—has been reported to have no significant impact on the incidence of neurological complications compared to deep hypothermic circulatory arrest (26, 27), and we consider it to be a reasonable strategy. Additionally, Lin et al. also reported that the short- and mid-term outcomes of patients who survived till hospital discharge were acceptable, including cerebral performance after discharge and a 3-year survival rate (12). Furthermore, preoperative CPA has been reported to be a significant risk factor for postoperative stroke (28); however, we believe the neurological outcome is less than hopeless. In addition, while the management strategy remains controversial for patients presenting with AAAD with a cerebrovascular accident or coma, a few studies have reported that early surgical intervention showed satisfactory recovery of consciousness and neurological function (29, 30). Therefore, neurological prognosis is not a justification for withdrawal of treatment in patients with AAAD and preoperative CPA, except in cases of apparent irreversible brain damage.

**Figure 1:**
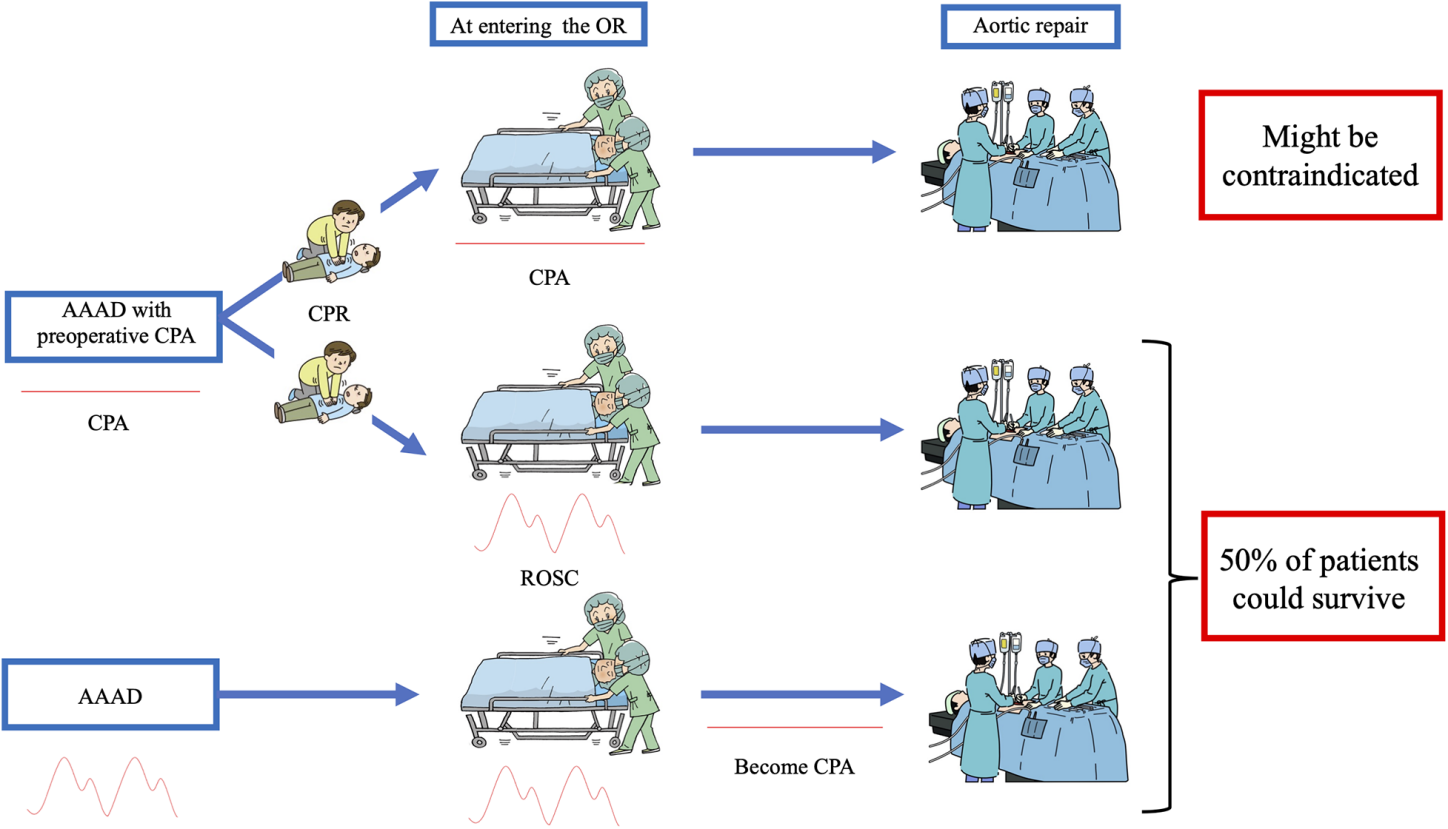
Decision tree applied at our hospital to treat patients with acute type A aortic dissection who had preoperative cardiopulmonary arrest (CPA). None of the patients without return of spontaneous circulation (ROSC) upon entering the operating room (OR) survived; therefore surgical treatment of such patients might be contraindicated. On the other hand, half of the patients who achieved ROSC upon entering the OR or who became CPA after entering the OR (i.e., patients who had spontaneous circulation upon entering the OR) survived, with acceptable neurological outcomes.

As described above, our institutional strategy for AAAD was a so called “all comer policy”, and we have aggressively performed aortic repair even in patients with ongoing and unsuccessful CPR. However, based on the results of the present study, we re-think about surgical indications in patients suffering from AAAD with preoperative CPA, and consider that surgery in patients undergoing CPR at the time of entering the OR might be contraindicated (Figure 1).

This study had some limitations. First, this was a single-center retrospective analysis of a limited number of patients. In the future, a multi-center analysis based on a large database may be desirable to confirm our conclusion. Second, the interval between the onset and start of surgery and the duration of CPR could not be analyzed. Third, this study focused only on short-term outcomes in a limited number of patients. Follow-up studies with more patients should be conducted to evaluate long-term outcomes.

In conclusion, the outcomes were extremely poor in patients with AAAD who had preoperative CPA and received ongoing CPR at entry into the OR. Therefore, surgical treatment might be contraindicated in such patients.

## Data Availability

The raw data supporting the conclusions of this article will be made available by the authors, without undue reservation.
